# Iron Deficiency Anemia among Hospitalized Children in Konya, Turkey

**DOI:** 10.1155/2013/514801

**Published:** 2013-12-18

**Authors:** Fatih Akin, Ece Selma Solak, Cengizhan Kilicaslan, Saltuk Bugra Boke, Sukru Arslan

**Affiliations:** ^1^Department of Pediatrics, Konya Training and Research Hospital, Meram, 42090 Konya, Turkey; ^2^Department of Pediatric Nephrology, Konya Training and Research Hospital, 42090 Konya, Turkey

## Abstract

The aim of this study was to investigate the characteristics of our hospitalized patients with the diagnosis of iron deficiency anemia (IDA) and effects of the IDA prevention project of the Turkish Ministry of Health which was started in 2004. The recommended dose of prophylactic iron supplementation was 1-2 mg/kg/day. The files of 1519 patients who were hospitalized to Konya Education and Research Hospital Pediatrics Clinic were reviewed. A total of 50 patients consisting of 35 boys and 15 girls with the mean age of 16,59 ± 1,68 months were included into the study. The prevalence of IDA was 3.29% (boys: 4.23%, girls: 2.1%). Hgb and Hct of the patients >24 months were significantly higher than those of the patients with the age of 6–12 months. Iron supplementation receiving rates were very low. Of the 28 patients older than 12 months, only 44% of them had received a full course of iron supplementation for 8 months. In conclusion, although prophylactic iron supplementation lowered the prevalences of IDA, receiving rates of iron supplementation were not adequate. While IDA is still a public health problem, prophylactic approaches should be carried out more effectively.

## 1. Introduction

Iron deficiency is the most common and widespread nutritional disorder in the world [1]. It is the only nutrient deficiency which is also significantly prevalent in all industrialized nations. According to the data of the World Health Organisation (WHO), the prevalence of iron deficiency anaemia (IDA) in industrialized countries and in nonindustrialized countries is 10–20% and 50–60%, respectively [2]. Globally, the prevalence of IDA in preschool-age children (0.00–4.99 years) and school-age children is 47.4% and 25.4%, respectively [3].

Iron deficiency impairs the cognitive development of children from infancy through adolescence. It also damages immune mechanisms and is associated with increased morbidity rates. The importance of iron deficiency and anaemia as a public health problem has been increasingly recognized by health authorities and policy makers. Although efforts are targeted primarily to prevent iron deficiency, it is still the most common nutrient deficiency all over the world [2].

The Turkish Ministry of Health started a project in 2004, which aimed to give 1-2 mg/kg daily prophylactic iron supplementation to all children aged 4 months until 1 year. In 2009, it was reported that after starting this project the prevalence of IDA had decreased [4]. In this paper we aimed to investigate the effects of the project after 8 years and characteristics of our hospitalized patients with IDA.

## 2. Materials and Methods

The files of 1519 patients who were hospitalized in Konya Training and Research Hospital Pediatrics Clinic between July 2012 and December 2012 for an acute illness were reviewed retrospectively. 50 patients were found to have the diagnosis of IDA. If diagnosis of anaemia was noticed, further evaluations including the measurements of serum iron, total iron-binding capacity (TIBC), and ferritin were performed. When indicated, vitamin B12, folic acid levels, and haemoglobin (Hgb) electrophoresis we obtained to rule out other nutrient deficiencies and haemoglobinopathies that can cause anaemia.

The patients included into the study were divided into 3 groups according to age: Group 1: 6–12 months, Group 2: 12–24 months, and Group 3: >24 months. WHO Hgb thresholds were used to identify patients as anaemic (Hgb < 11 g/dL for patients 6–59 months old, Hgb < 11.5 for patients >59 month old). IDA was defined as Hgb values less than WHO thresholds with the presence of two or more of the following parameters; mean corpuscular volume (MCV) less than 70 fl, serum ferritin below 30 mcg/L, and transferrin saturation (TSAT) less than 16% [5, 6]. Patients having chronic illness, hematologic diseases, and thalassemia traits or receiving any chronic medication were not enrolled into the study. The variables examined in the study were Hgb, haematocrit (Hct), MCV, mean corpuscular haemoglobin (MCH), mean corpuscular haemoglobin concentration MCHC, red blood cell (RBC), red cell distribution width (RDW), white blood cell (WBC), platelet (Plt), iron, TIBC, TSAT, and ferritin levels of the patients.

### 2.1. Statistical Analysis

The statistical analysis was carried out using the SPSS 15.0 statistical software package for Windows. Descriptive statistics of continuous variables were expressed as mean ± standard deviation (sd). Independent samples *t*-test was used to compare the mean values of the groups. Mann Whitney-*U* test was used for nonnormally distributed variables (Iron and TSAT). When comparing the means of more than two groups, one-way ANOVA and Kruskal-Wallis test were conducted. If there was difference between groups, Tukeys test was performed to compare between the groups  *P* < 0.05 was considered as significance level.

## 3. Results

A total of 50 patients were included into the study (35 boys and 15 girls). The age range of the patients was from 6 months to 80 months (mean age 16,59 ± 1,68 months). Mean age for boys and girls was 14,87 ± 1,41 and 20,56 ± 4,51 months, respectively.

Among a total of 1519 hospitalized patients, the prevalence of IDA was 3.29% (boys: 4.23%, girls: 2.1%). Mean values (mean ± sd) of Hgb, Hct, MCV, MCH, MCHC, RBC, RDW, WBC, Plt, iron, TIBC, ferritin, and TSAT according to sex are given in Table 1. There was no significant difference between girls and boys according to the variables except WBC. WBC levels of girls were significantly higher (*P* < 0.05).

RDW levels were significantly higher in patients aged between 13 and 24 months (17.66 ± 0.41) than the patients >24 months (15.61 ± 0.21).

With respect to age, the number of the subjects in Group 1, Group 2 and Group 3 was, 22, 21, and 7, respectively. Hgb levels were gradually increasing with age and this association was statistically significant (Table 2). Hgb and Hct of the patients >24 months old were significantly higher than those of the patients with the age of 6–12 months (*P* < 0.05). Additionally, when mean Hgb levels were compared with the age-appropriate Hgb thresholds, the levels of Groups 1 and 2 were significantly lower than those of the thresholds.

Although prophylactic iron supplementation is recommended to be used from 4 months until 1 year, there were patients who never received or received only one bottle (a usage of nearly 1 month) of iron supplementation. Of the 28 patients older than 12 months, only 28% (8 patients; 6 boys, 2 girls) received full course of iron supplementation for 8 months, where 28% (8 patients; 7 boys 1 girl) of them receive it only for 1 month. The rest 44% did not receive iron supplementation despite the recommendations of their family physicians. Although rate of prophylactic iron supplementation use was significantly decreasing with age, there was no significant difference among variables according to receiving iron supplementation.

## 4. Discussion

Iron deficiency is the most widespread and common nutritional disorder in the world inspite of the efforts to decrease the frequency. The prevalence varies in different parts of the world with higher rates in the developing countries [5, 7]. The results of studies from different provinces of Turkey also showed that iron deficiency is the most important cause of anaemia. Koçak et al. reported that the prevalence of IDA was 17.2% with the highest prevalence in infants being 48% in southern Turkey [8]. Another study conducted in the western part of Turkey reported that the prevalence of IDA was 6.5% among adolescent age [9]. Kilinç et al. reported that the prevalence of IDA in the Southeastern Anatolia of Turkey was 15.5% among 2–5-year-old children [10]. Cetinkaya et al. investigated severe IDA among hospitalized children aged 7–24 months and reported that 61.6% of 3117 children had anaemia and 2.7% of them were severe IDA [11]. Comparing with these studies, the prevalence of IDA which is 3.29% is lower in our study. This may be due to the iron supplementation project of the health ministry started in 2004, as those studies were conducted before 2004.

WHO recommends prophylactic supplementation of iron at a dosage of 2 mg/kg/day to all children between 6 and 23 months of age, especially where the diet does not include fortified foods, or prevalence of anaemia in children approximately 1 year of age is severe [2]. The project of the Turkish Ministry of Health aimed to give iron supplementation to all children aged 4 months until 1 year. After 5 years in 2009, the ministry reported that prevalence of IDA had decreased to 7.8% from 15.2–62.5% prevalence rates, but IDA was still a public health problem in Turkey [4]. The low prevalence examined in our study may suggest that prophylactic iron supplementation has gradually increased. From the finding that the Hgb levels of patients aged 6–24 months were significantly lower than those of the age-appropriate thresholds, it can be considered that extension of prophylactic iron supplementation duration to 24 months might be more favorable.

Ferritin is an intracellular hollow protein shell composed of 24 subunits surrounding an iron core that may contain as many as 4000–4500 iron atoms. In the body, small amounts of ferritin are secreted into the plasma. The concentration of this plasma ferritin is positively correlated with the size of the total body iron stores in the absence of inflammation. Low serum ferritin values reflects depleted iron stores [12]. Ferritin levels, in contrast to Hgb, are not affected by residential elevation above sea level or smoking behaviour. However, ferritin is a positive acute phase response protein whereby concentrations increase during an inflammatory disease or subclinical infection. This makes the interpretation of normal or high serum ferritin values difficult in areas of widespread infection or inflammation [2]. The cutoff values of ferritin were revised in 1993 and it was reported that ferritin levels < 12 *μ*g/L under 5 years of age, <15 *μ*g/L above 5 years of age and <30 *μ*g/L in the presence of infection reflect the depleted iron stores [12]. In the study of Phiri conducted on patients with severe anaemia in a high infection pressured area, they found mean ferritin levels to be 729.2 *μ*g/L and suggested that it was necessary to change the cutoff limit of ferritin from 30 to 273 *μ*g/L in order to improve its diagnostic efficiency [13]. The mean ferritin level of our patients was 30.71 ± 4.58 *μ*g/L (min.: 2.4; max.: 179.4). 34% of our subjects had a ferritin level above 30 mcg/L with other parameters indicating IDA. All of these subjects had an accompanying inflammatory disease.

Anaemia is a late manifestation of iron deficiency, and iron deficiency without anaemia is even more widespread. If subtle effects of iron deficiency in infancy lay the ground for later problems in cognitive and behavioral functioning, then a large unrecognized population of children could be at risk due to perinatal iron deficiency, a nutritional problem that can be prevented or treated [14]. Consequences of IDA during childhood include growth retardation, reduced school achievement, impaired motor and cognitive development, and increased morbidity from infections including especially diarrhea and acute respiratory infections [2]. Specifically, iron deficiency can lead to deficits in memory and behavioural regulation as iron is required to make neurotransmitters such as dopamine, epinephrine, and serotonin while impaired myelination contributes to deficits in motor function [15–17]. Some of these impairments are thought to be irreversible if they occur at an early age and the consequences may continue even after treatment, reinforcing the importance of prevention [15, 18, 19].

## 5. Conclusions

In conclusion, although prophylactic iron supplementation lowered the prevalences of IDA, IDA is still a public health problem throughout the world. Iron deficiency impairs the cognitive development of children from infancy through adolescence. Therefore, prophylactic approaches should be carried out more effectively. It can be considered that extension of prophylactic iron supplementation duration to 24 months might be more favorable in our country. Because ferritin levels are increased during inflammatory processes, the cutoff levels should be reevaluated.

## Figures and Tables

**Table 1:** Laboratory characteristics of patients with IDA.

	Boys (*n* = 35)	Girls (*n* = 15)	*P*

Hgb (g/dL)	9.77 ± 0.14	9.69 ± 0.34	>0.05
Hct (%)	31.03 ± 0.48	31.37 ± 0.83	>0.05
MCV (fL)	6496 ± 0.87	64.13 ± 1.82	>0.05
MCH (pg)	20.50 ± 0.39	19.79 ± 0.73	>0.05
MCHC (gr/dL)	31.48 ± 0.25	30.79 ± 0.47	>0.05
RBC (M/mL)	4.81 ± 0.10	4.93 ± 0.18	>0.05
RDW (%)	16.75 ± 0.31	17.36 ± 0.57	>0.05
WBC (*µ*L^−1^)	10.81 ± 0.63	13.75 ± 1.56	<0.05
PLT (*µ*L^−1^)	384742.85 ± 22314.90	408000.00 ± 38686.28	>0.05
Iron (*µ*g/dL)	29.17 ± 4.88	18.86 ± 2.71	>0.05
TIBC (*µ*g/dL)	339.14 ± 18.48	345.73 ± 16.63	>0.05
Ferritin (*µ*g/L)	29.43 ± 5.66	33.68 ± 7.87	>0.05
TSAT (%)	15.93 ± 6.65	5.40 ± 0.72	>0.05

**Table 2:** IDA parameters of the patients with respect to age.

Age	*N*	Hgb (g/dL)	MCV (fL)	Ferritin (*µ*g/L)	TSAT (%)
6–12 months	22	9.40 ± 0.15	65.20 ± 1.17	25.44 ± 4.25	10.79 ± 3.20
12–24 months	21	9.81 ± 0.26	64.20 ± 1.17	29.94 ± 8.55	6.04 ± 0.67
>24 months	7	10.61 ± 0.22	64.72 ± 3.01	49.54 ± 14.83	39.19 ± 32.06

## References

[1] DeMaeyer E, Adiels-Tegman M (1985). The prevalence of anaemia in the world. *World Health Statistics Quarterly*.

[2] World Health Organisation (2001). *Iron Deficiency Anaemia Assessment, Prevention, and Control*.

[3] World Health Organisation (2008). *Worldwide Prevalence of Anaemia 1993–2005*.

[4] Turkish Ministry of Health (2009). *The Report of the Study of Iron Usage among 12–23 Month-Old Children*.

[5] Lerner NB, Sills R, Kliegman RM, Stanton BF, Geme JSt, Schor N, Behrman RE (2011). Iron deficiency anemia. *Nelson Textbook of Pediatrics*.

[6] Male C, Persson LÅ, Freeman V, Guerra A, Van’t Hof MA, Haschke F (2001). Prevalence of iron deficiency in 12-mo-old infants from 11 European areas and influence of dietary factors on iron status (Euro-Growth study). *Acta Paediatrica*.

[7] World Health Organisation (1989). *Preventing and Controlling Iron Deficiency Anaemia through Primary Health Care*.

[8] Koçak R, Alparslan ZN, Ağridağ G, Başlamisli F, Aksungur PD, Koltaş S (1995). The frequency of anaemia, iron deficiency, hemoglobin S and beta thalassemia in the south of Turkey. *European Journal of Epidemiology*.

[9] Aydınok Y, Öztop Ş, Nişli G, Kavaklı K, Çetingül N (1998). Percentile norms and curves for hematological values in Turkish adolescents. *Turkish Journal of Haematology*.

[10] Kilinç M, Yüregir GT, Ekerbiçer H (2002). Anaemia and iron-deficiency anaemia in south-east Anatolia. *European Journal of Haematology*.

[11] Cetinkaya F, Yildirmak Y, Kutluk G (2005). Severe iron-deficiency anemia among hospitalized young children in an urban hospital. *Pediatric Hematology and Oncology*.

[12] World Health Organisation (2011). *Serum Ferritin Concentrations for the Assessment of Iron Status and Iron Deficiency in Populations*.

[13] Phiri KS, Calis JCJ, Siyasiya A, Bates I, Brabin B, Boele van Hensbroek M (2009). New cut-off values for ferritin and soluble transferrin receptor for the assessment of iron deficiency in children in a high infection pressure area. *Journal of Clinical Pathology*.

[14] Lozoff B (2000). Perinatal iron deficiency and the developing brain. *Pediatric Research*.

[15] Iannotti LL, Tielsch JM, Black MM, Black RE (2006). Iron supplementation in early childhood: health benefits and risks. *American Journal of Clinical Nutrition*.

[16] Moy RJD (2006). Prevalence, consequences and prevention of childhood nutritional iron deficiency: a child public health perspective. *Clinical and Laboratory Haematology*.

[17] Beard JL (2008). Why iron deficiency is important in infant development. *Journal of Nutrition*.

[18] Lozoff B (2007). Iron deficiency and child development. *Food and Nutrition Bulletin*.

[19] Siddiqui IA, Rahman MA, Jaleel A (2004). Efficacy of daily vs. weekly supplementation of iron in schoolchildren with low iron status. *Journal of Tropical Pediatrics*.

